# Activation of primary hepatic stellate cells and liver fibrosis induced by targeting TGF-β1/Smad signaling in schistosomiasis in mice

**DOI:** 10.1186/s13071-022-05584-1

**Published:** 2022-12-06

**Authors:** Ping Huang, Huihui Ma, Yun Cao, Tingzheng Zhan, Tingting Zhang, Xinyi Wang, Yanan Zhang, Jing Xu, Chaoming Xia

**Affiliations:** 1 Department of Pathogen Biology, Suzhou Medical College of Soochow University, 199 Renai Road, Suzhou, 215123 Jiangsu China; 2 Department of Laboratory Medicine, Bengbu Medical College, 2600 Donghai Road, Bengbu, 23303 Anhui China; 3 Department of Pathogen Biology, Guangxi Medical University, 22 Shuangyong Road, Nanning, 530021 Guangxi China

**Keywords:** Schistosomiasis, Liver fibrosis, Hepatic stellate cells, TGF-β1/Smad signaling, MicroRNAs

## Abstract

**Background:**

In mice, liver fibrosis is the most serious pathologic change during *Schistosoma japonicum* (*S. japonicum*) infection. Schistosomiasis is mainly characterized by schistosome egg-induced granulomatous fibrosis. Hepatic stellate cells (HSCs) are mainly responsible for the net accumulation of collagens and fibrosis formation in the liver. Activated HSCs regulated by transforming growth factor-β1 (TGF-β1)/Smad signaling have emerged as the critical regulatory pathway in hepatitis virus or carbon tetrachloride-induced liver fibrosis. However, the detailed mechanism of HSC activation in schistosome-induced liver fibrosis is poorly understood.

**Methods:**

*Schistosoma japonicum*-induced murine models and a control group were generated by abdominal infection with 15 (± 1) cercariae. The purity of cultured primary HSCs was evaluated by immunocytochemistry. The histopathological changes in the livers of infected mice were estimated by hematoxylin–eosin and Masson staining. Dynamic expression of pro-fibrotic molecules and microRNAs was detected by real-time quantitative PCR (RT-qPCR). Mainly members involved in the TGF-β1/Smad signaling pathway were examined via RT-qPCR and Western blot.

**Results:**

The egg-induced granulomatous inflammation formed at 4 weeks post-infection (wpi) and developed progressively. Alpha-smooth muscle actin (α-SMA), collagen I, collagen III, TGF-β1, Smad2, Smad3, and Smad4 showed a significant increase in mitochondrial RNA (mRNA) and protein expression compared with the control group at 7 and 9 weeks post-infection (wpi), while an opposite effect on Smad7 was observed. In addition, the mRNA expression of microRNA-21 (miRNA-21) was significantly increased at 7 wpi, and the mRNA expression of miRNA-454 was decreased starting from 4 wpi.

**Conclusion:**

Our present findings revealed that HSCs regulated by the TGF-β1/Smad signaling pathway play an important role in liver fibrosis in *S. japonicum*-infected mice, which may provide proof of concept for liver fibrosis in schistosomiasis.

**Graphical Abstract:**

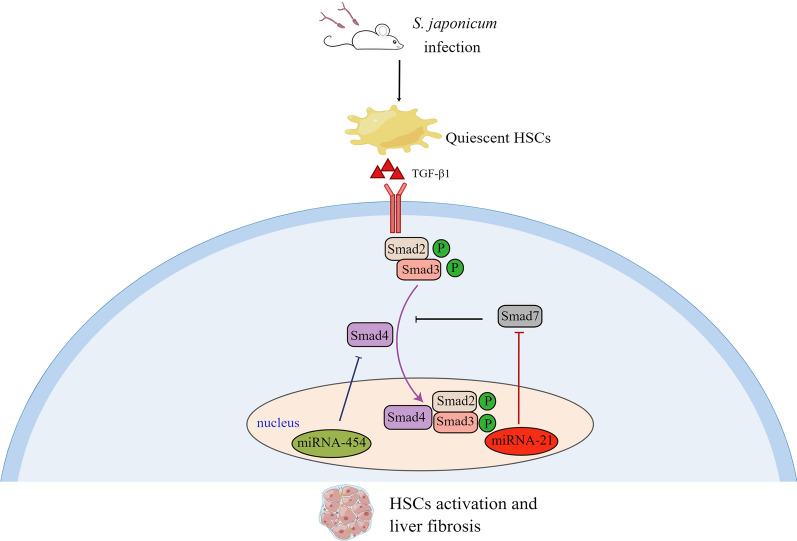

**Supplementary Information:**

The online version contains supplementary material available at 10.1186/s13071-022-05584-1.

## Background

Schistosomiasis is a serious zoonotic parasitic disease caused by trematode worms of the genus *Schistosoma* and is classified as a neglected tropical disease by the World Health Organization (WHO). Among the six species of *Schistosoma*, of which *S. japonicum*, *S. mansoni*, and *S. haematobium* are the most clinically relevant species, whereas *S. mekongi*, *S. intercalatum,* and *S. guineensis* have lower prevalence [1]. According to WHO, over 230 million people worldwide are infected with *Schistosoma* spp., causing approximately 200,000 deaths annually [2]. It is the second most common parasitic disease worldwide after malaria in terms of high prevalence and risk, resulting in more than 70 million disability-adjusted life years [3]. People become infected with schistosomiasis when their skin comes into contact with water containing cercariae larvae released by freshwater snails. In the body, cercariae develop from juveniles into adults. Adult worms eventually settle in blood vessels, where the female worms produce hundreds to thousands of eggs per day [4]. Some eggs are passed out in the host’s feces or urine to continue the next life-cycle, and others are deposited in body tissues such as the liver, small intestine (for *S. mansoni*, *S. japonicum*, and *S. mekongi*), and bladder (for *S. haematobium*), causing an imbalance in immunoregulation and progressive organ lesions [5, 6].

Liver fibrosis is a wound-healing process associated with chronic liver injury caused by the hepatitis virus, helminth infections, nonalcoholic steatohepatitis (NASH), and metabolic and autoimmune diseases [7–10]. Previous studies have demonstrated that schistosome eggs, not adult worms, are mainly responsible for inducing morbidity and mortality associated with schistosomiasis. The eggs induce eosinophils, basophils, and mast cells, elevate immunoglobulin E (IgE) levels, suppress inflammatory responses, and contribute to granuloma formation and liver fibrosis [1]. All evidence suggests that activated hepatic stellate cells (HSCs) are the most central cellular players during liver fibrosis. Generally, when the liver is subjected to chronic injury, quiescent HSCs transition to activated HSCs. This accelerates the formation of fibrosis associated with the expression of a pro-fibrogenic gene such as transforming growth factor beta 1 (TGF-β1), alpha-smooth muscle actin (α-SMA), collagen I and III, excessive synthesis, and less degradation of extracellular matrix (ECM) [11–13].

The TGF-β-mediated pathogenic mechanism plays an important role in tissue fibrosis. The intracellular signaling of TGF-β1 is regulated by Smad proteins including Smad2, Smad3, Smad4, and Smad7 [14]. TGF-β1 directly activates Smad signaling through interaction with the TβRII cell surface receptors. Then, signal transduction from TβRI to the nucleus, resulting in phosphorylation of Smad2 and Smad3. Subsequently, the p-Smad2, p-Smad3, and Smad4 form oligomer complexes that transfer to the nucleus and regulate the transcription of target genes [14]. In the context of liver fibrosis, Smad7 acts as a negative feedback regulator, inhibiting the TGF-β1/Smad signaling by preventing the binding of activated TβRI to Smad2/Smad3 [15]. MicroRNAs (miRNAs) are endogenous, small non-coding RNAs that bind to the 3′ untranslated region (3′-UTR) of specific mitochondrial RNAs (mRNAs) and cause either blockade of translation or mRNA degradation [16]. Previous studies have indicated that miRNAs are associated with the development of liver fibrosis. Downregulation of miRNA-21 in HSCs attenuated liver fibrosis through overexpression of Smad7 [17, 18]. Moreover, miRNA-454 directly targeted Smad4 to inhibit HSC activation [19]. However, the specific regulatory mechanism of HSC has rarely been investigated in the complicated pathogenic process of schistosomiasis.

Therefore, we attempted to investigate the area of change of egg granulomas and the activation of the primary HSCs in liver fibrosis to further reveal the molecular mechanisms of schistosomiasis.

## Methods

### Animals

Female FVB/NJ mice (6–8 weeks old) were purchased from the Center of Comparative Medicine of Yangzhou University. All mice were kept under specific pathogen-free conditions at the laboratory animal research facility of Soochow University. *Schistosoma japonicum-*harboring *Oncomelania hupensis* was supplied by the Jiangsu Institute of Parasitic Diseases, Wuxi, China. *Oncomelania hupensis* were carefully raised in a Petri dish containing wet papyrus.

### Mice model

*Oncomelania hupensis* was placed in chlorine-free water under an incandescent lamp for 2 h to release cercariae. The abdomens of mice were shaved and moistened with dechlorinated water to infect mice with cercariae. Then, 15 ± 1 cercariae were placed on a cover slide and attached to the abdominal skin for 20 min. At 4, 7, and 9 weeks post-infection (wpi), mice with or without infection were sacrificed under anesthesia with 2% pentobarbital, and the livers were obtained for further studies.

### Isolation and culture of primary murine HSCs

HSCs were isolated from the livers of FVB/NJ mice according to modified procedures described previously [20]. In brief, mice were euthanized and soaked in ethanol for 45 s for sterilization, after which scissors were used to cut the abdominal skin, exposing the liver and heart. Preheated RPMI 1640 (Sigma-Aldrich, St. Louis, MO, USA) was injected into the liver through the hepatic portal vein and drained via the inferior vena cava with a peristaltic pump. When the blood was washed clean, preheated RPMI 1640 (Gibco, CA, USA) containing 0.04% collagenase I was pumped into the liver for 6 min at a flow rate of 15 ml/min. Then the connective tissue and adipose tissue of the liver were removed, ground, and digested at 39 °C with 20 ml RPMI 1640 containing 0.08% pronase E (Solarbio), 0.08% collagenase I (Solarbio), and 5 U/ml DNase I (Solarbio). After 15 min, the digestion was terminated immediately by adding 20 ml RPMI 1640 and the solution filtered using a 70 μm membrane into a 50 ml tube. The supernatant was centrifuged at 4 °C and 400 g for 6 min to remove the hepatocytes and washed twice with RPMI 1640. The cell pellet was resuspended in 5 ml 15% OptiPrep™ (Axis-Shield, Oslo, Norway) and 5 ml 11.5% OptiPrep, and then gently overlaid onto 2 ml RPMI 1640. After centrifugation at 1400 g for 17 min without braking, purified HSCs were obtained. HSCs were cultured at a density of 2 × 10^6^ cells/well in Dulbecco's modified Eagle medium (DMEM) supplemented with 15% premium fetal bovine serum (Gibco), 100 U/ml penicillin, and 100 μg/ml streptomycin, and then placed in an incubator (37 °C, 5% CO_2_).

### Immunocytochemistry staining

Primary HSCs were cultured for 3 days and then collected and fixed using 4% paraformaldehyde for 60 min. The sections were blocked with 10% normal goat serum (Boster, Wuhan, China) in phosphate-buffered saline (PBS) at room temperature for 30 min ~ 1 h to inhibit unspecific binding and incubated with primary glial fibrillary acidic protein (GFAP) polyclonal antibody (booster) in antibody diluent at 4 °C for 12 h or overnight. After the sections were washed three times with PBS, the secondary antibody of biotin-labeled goat anti-rabbit IgG was added to each section and incubated at room temperature for 30 min. Then, the sections were stained with SABC-Cy3 for 30 min and washed three times with PBS. For immunocytochemistry analysis, the sections were mounted with Vectashield mounting medium (Millipore, Burlington, MA, USA) and visualized by a microscope (Olympus, Tokyo, Japan). Finally, 10 digital images of each section were randomly selected, and the ratio of GFAP^+^ cells was calculated.

### Histopathology

Mice were sacrificed, and liver tissues were collected at 0, 4, 7, and 9 weeks and fixed in 4% paraformaldehyde. The liver tissues were embedded in paraffin and stained with hematoxylin–eosin (H&E) or Masson’s Trichrome according to the manufacturer’s protocol. The pathological changes in each section were observed under an upright microscope (Olympus) and measured using Image-Pro Plus 6.0 software (Media Cybernetics Inc., MD, USA). To assess the degree of hepatic fibrosis at different time points post-infection with *S. japonicum*, at least five mice were analyzed at each point.

### RNA extraction, complementary DNA (cDNA) synthesis, and real-time quantitative polymerase chain reaction (RT-qPCR)

Total RNA was extracted from primary HSCs with TRIzol reagent (Thermo Fisher Scientific, Waltham, MA, USA) following the manufacturer’s protocol. Then, the concentration and purity of total RNA were examined using the NanoDrop 2000c spectrophotometer (Thermo Fisher Scientific). After RNA detection was qualified, the first-strand cDNA was synthesized from RNA using the Prime-Script™ RT reagent Kit (Takara, Tokyo, Japan). The expression levels of liver fibrosis-related genes were detected using the SYBR Premix Ex Taq RT-PCR Kit (Takara, Tokyo, Japan), and the specific primer sequences of genes are displayed in Table 1. β-actin served as the internal reference gene. The PCR reaction was run on the 7500 Real-Time PCR System (Thermo Fisher Scientific) at 95 °C for 10 min, followed by 40 cycles at 95 °C for 15 s and 60 °C for 1 min. Then, the melting curve was measured (95 °C for 30 s, 65 °C for 15 s, and 95 °C for 30 s). The experiments were performed as independent biological replicates at least three times, and the relative expression of the target genes was calculated using the 2^−ΔΔCt^ method.

**Table 1:** Primers to amplify the liver fibrosis-related genes (mice)

Gene	Forward primer sequence	Reverse primer sequence
Collagen I	CCTGGCAAAGACGGACTCAAC	GCTGAAGTCATAACCGCCACTG
Collagen III	CTGTAACATGGAAACTGGGGAAA	CCATAGCTGAACTGAAAACCACC
α-SMA	GTCCCAGACATCAGGGAGTAA	TCGGATACTTCAGCGTCAGGA
TGF-β1	GTGCGGCAGCTGTACATTGACTTT	TGTGTTGGTTGTAGAGGGCAAGGA
Smad2	ATGTCGTCCATCTTGCCATTC	AACCGTCCTGTTTTCTTTAGCTT
Smad3	GTCAACAAGTGGTGGCGTGTG	GCAGCAAAGGCTTCTGGGATAA
Smad4	TGACGCCCTAACCATTTCCAG	CTGCTAAGAGCAAGGCAGCAAA
Smad7	AGAGGCTGTGTTGCTGTGAATC	CCATTGGGTATCTGGAGTAAGGA
β-actin	CGCTGTATTCCCCTCCATCG	CCAGTTGGTAACAATGCCATGT
miRNA-21	GCGGCGGTAGCTTATCAGACT	
miRNA-454	GCGGCGGTAGTGCAATATTGC	

### Western blot

The total protein of HSCs was extracted using RIPA lysis buffer (Thermo Fisher Scientific) containing 1% protease and phosphatase inhibitor cocktail (Thermo Fisher Scientific) and quantified with a BCA protein assay kit (Beyotime, Shanghai, China). A 30 μg protein was electrophoresed on 5% or 10% sodium dodecyl sulfate polyacrylamide gel electrophoresis (SDS-PAGE) and transferred to polyvinylidene fluoride (PVDF) membranes with 100 mA for 2 h. The membranes were incubated with tris-buffered saline (TBS)/Tween 20^®^ (TBST) containing 5% non-fat milk at room temperature for 1.5 h to avoid nonspecific binding of epitopes and incubated with the following primary antibodies overnight at 4 °C: anti-α-SMA (Abcam, Cambridge, UK), anti-collagen I (Bioss, Beijing, China), anti-collagen III (Proteintech, Chicago, IL, USA), anti-TGF-β1(Abcam), anti-Smad2/3 (Abcam), anti-p-Smad2/3 (Cell Signaling Technology, Danvers, MA, USA), anti-Smad4 (Abcam), anti-Smad7 (Abcam), and anti-β-actin (Bioss) was used for normalization. After washing three times with TBST, the membranes were incubated with horseradish peroxidase (HRP)-conjugated goat anti-rabbit secondary antibodies at room temperature for 30 min. The protein bands were visualized using a super-sensitive chemiluminescence reagent (Meilun, Dalian, China) on a chemiluminescence imaging system (Bio-Rad, CA, USA). The density of the protein bands was measured using ImageJ2x software, following the manufacturer’s instructions.

### miRNA target prediction

TargetScan (http://www.targetscan.org) [21] and MiRanda (http://www.microrna.org/microrna/home.do) [22] were used to predict possible target genes of miRNAs and conserved sites among different species.

### Statistical analysis

Data are shown as the mean ± standard deviation (SD). The statistical analyses were performed using SPSS 21.0 Data Editor (IBM Corporation, Armonk, NY, USA). Differences between the two groups were determined with unpaired, two-tailed Student’s *t*-tests. Multiple groups were analyzed by one-way analysis of variance (ANOVA) followed by the Tukey–Kramer test. A value of *P* < 0.05 was considered statistically significant.

## Results

### Histopathological observation of the livers of *S. japonicum*-infected mice

**Fig. 1:**
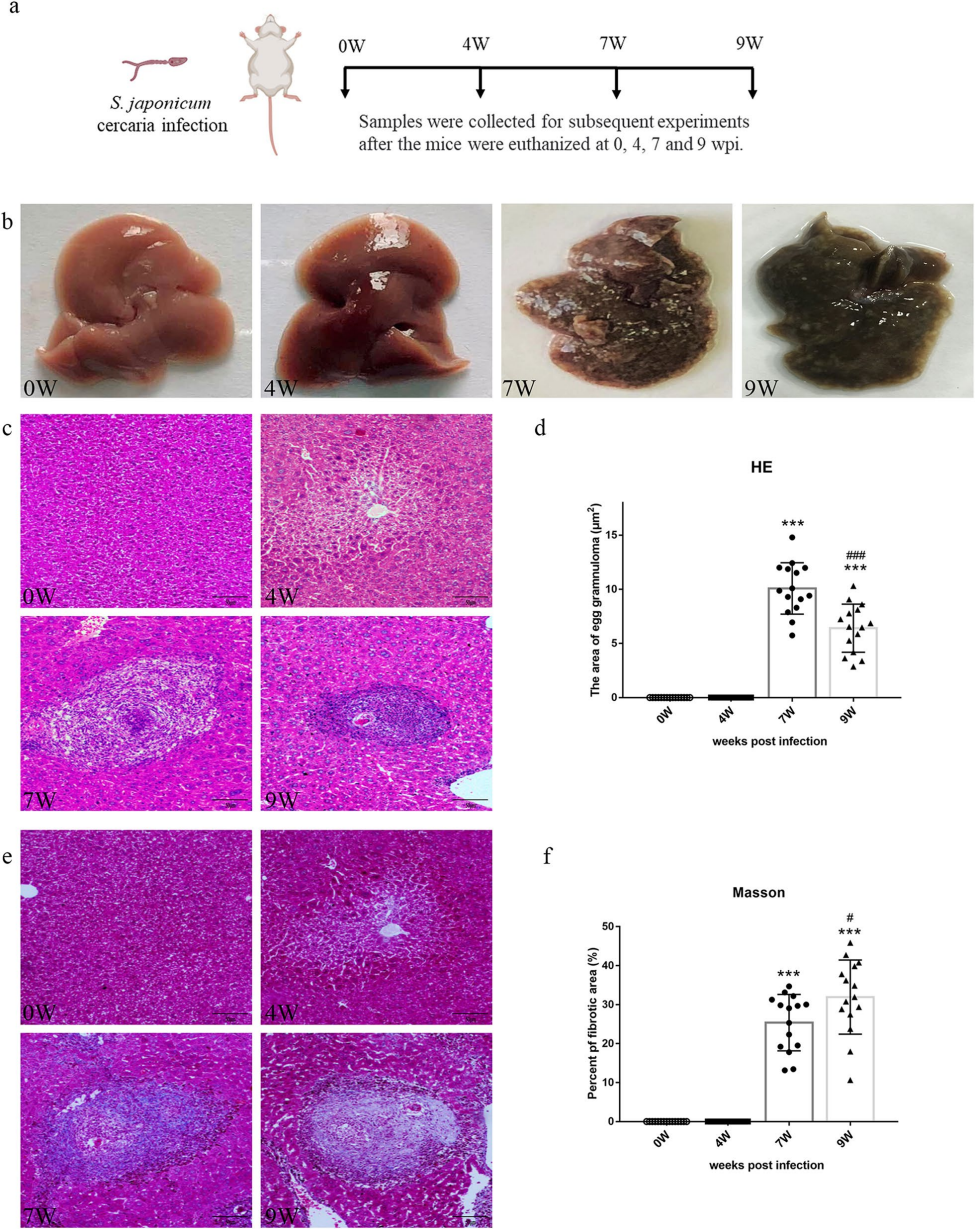
Histopathological alterations in *S. japonicum*-infected liver at 0, 4, 7, and 9 weeks. **a** Schematic diagram of mice infected with *S. japonicum* cercariae and experiments conducted at different time points after infection. **b** Anatomy photographs of the liver were taken with a camera. **c** Representative images of egg granuloma stained by H&E in paraffin-embedded liver tissues (scale bars:100 μm). **d** The area of egg granuloma in the liver was measured using Image-Pro Plus 6.0. **e** Representative images of collagen deposition stained with Masson’s trichrome in paraffin-embedded liver tissues (scale bars:100 μm). **f** The percentage of collagen area was observed by Image-Pro Plus 6.0. Data are presented as mean ± SD (*n* = 15). ***P < 0.001, compared with the 0 wpi group. ^#^*P* < 0.05, ^###^*P* < 0.001 compared with the 7 wpi group

To evaluate the histopathological changes in the mice infected with *S. japonicum* mice, histological examination of the livers was performed using H&E and Masson’s staining at different stages after infection (Fig. 1a). In mice livers at 0 and 4 weeks post-infection (wpi), tissue injuries were invisible, while the livers at 7 and 9 wpi exhibited a gray-black color and had numerous irregular nodules on the surface (Fig. 1b). In mice livers at 0 and 4 wpi, H&E staining showed the complete structure of hepatic lobules and ruled arrangement of hepatic cells, and egg granulomas formed by a large number of worm eggs and surrounded inflammatory cells at 7 and 9 wpi (Fig. 1c). Furthermore, the area of egg granuloma in the livers at 7 wpi was significantly higher versus the mice at 9 wpi (7 wpi: 10.08 ± 2.38 versus 9 wpi: 6.41 ± 2.22, *P* < 0.001) (Fig. 1d). Meanwhile, the Masson staining revealed that a large number of blue-stained collagen fibers were deposited in the liver tissue of mice at 7 and 9 wpi, especially deposited around egg granulomas (Fig. 1e). The fibrotic area in total liver sections was 25.38 ± 7.23 at 7 wpi, significantly lower versus the mice at 9 wpi (31.92 ± 9.51, *P* < 0.05), indicating that as the infection time increased, the increase in collagen fibers in mice liver and their fibrosis status became more serious.

### Detection of the dynamic expression of pro-fibrotic molecules in primary HSCs

**Fig. 2:**
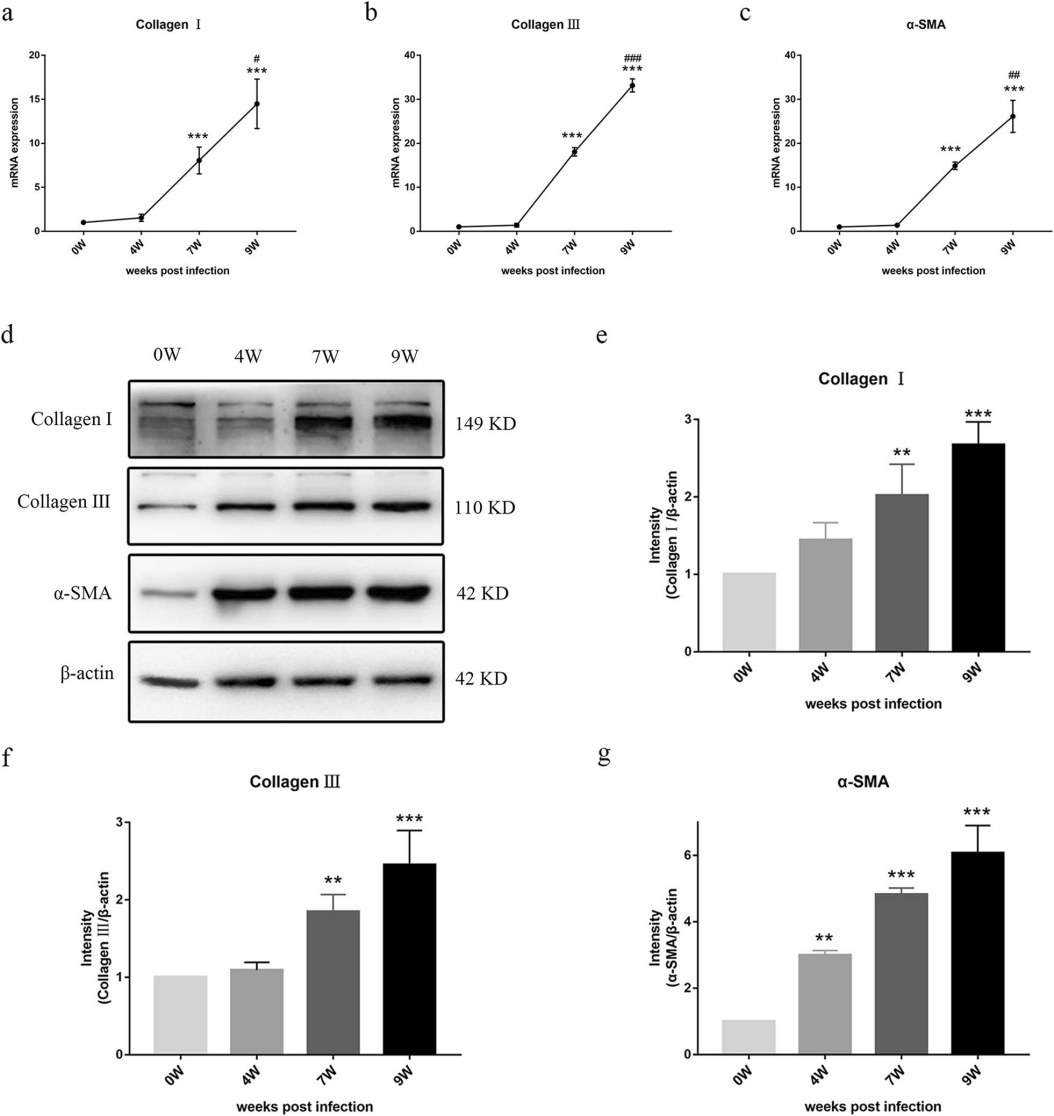
The expression of α-SMA, collagen I, and collagen III in primary HSCs of *S. japonicum*-infected mice at 0, 4, 7, and 9 weeks. **a**–**c** Messenger RNA levels of α-SMA, collagen I, and collagen III were determined by RT-qPCR. **d** The expression levels of α-SMA, collagen I, and collagen III were evaluated by Western blot (**e**–**g**), and the densitometric analysis was normalized to the level of endogenous control (β-actin) in primary HSCs. Data are presented as mean ± SD (*n* = 3). *P < 0.01, ***P < 0.001, compared with the 0 wpi group. ^#^*P* < 0.05, ^##^*P* < 0.01, ^###^*P* < 0.001 compared with the 7 wpi group

We next isolated the primary HSCs from the livers of *S. japonicum*-infected mice at different time points (0, 4, 7, 9 wpi). HSCs have three major characteristics: star-like shape, spontaneous fluorescence excited by ultraviolet of 328 nm wavelength, and specific expression of GFAP. According to these criteria, the purity of the obtained HSCs was over 90% (Additional file 1: Fig. S1). To examine the dynamic expression of pro-fibrotic molecules in primary HSCs and the synthesis of ECM proteins in response to *S. japonicum* infection, we measured the mRNA and protein levels of α-SMA and collagen I and III in primary HSCs. Our RT-qPCR assays showed that the upregulation of α-SMA and collagen I and III was observed at 7 wpi and peaked at 9 wpi, with an approximately 26.12-fold change in α-SMA (*P* < 0.001), 14.48-fold change in collagen I (*P* < 0.001) and 33.15-fold change in collagen III (*P* < 0.001) (Fig. 2), compared with the primary HSCs at 0 wpi. In addition, we detected the expression levels of proteins associated with liver fibrosis by Western blot. Consistent with the results of RT-qPCR, the protein levels of α-SMA and collagen I and III were also increased as the disease progressed, peaking at 9 wpi. The protein levels of α-SMA and collagen I and III in the HSCs at 9 wpi were 6.08-fold (*P* < 0.001), 2.67-fold (*P* < 0.001), and 2.46-fold (*P* < 0.001) higher than those of HSCs at 0 wpi, respectively. Interestingly, we found there was no significant effect in the protein expression of pro-fibrotic molecules between the 7 wpi and 9 wpi groups (*P* > 0.05) (Fig. 2). Our findings demonstrated that activation of HSCs occurred in the mice liver after being infected with *S. japonicum*.

### Determination of the effect of TGF-β1/Smad signaling in primary HSCs

**Fig. 3:**
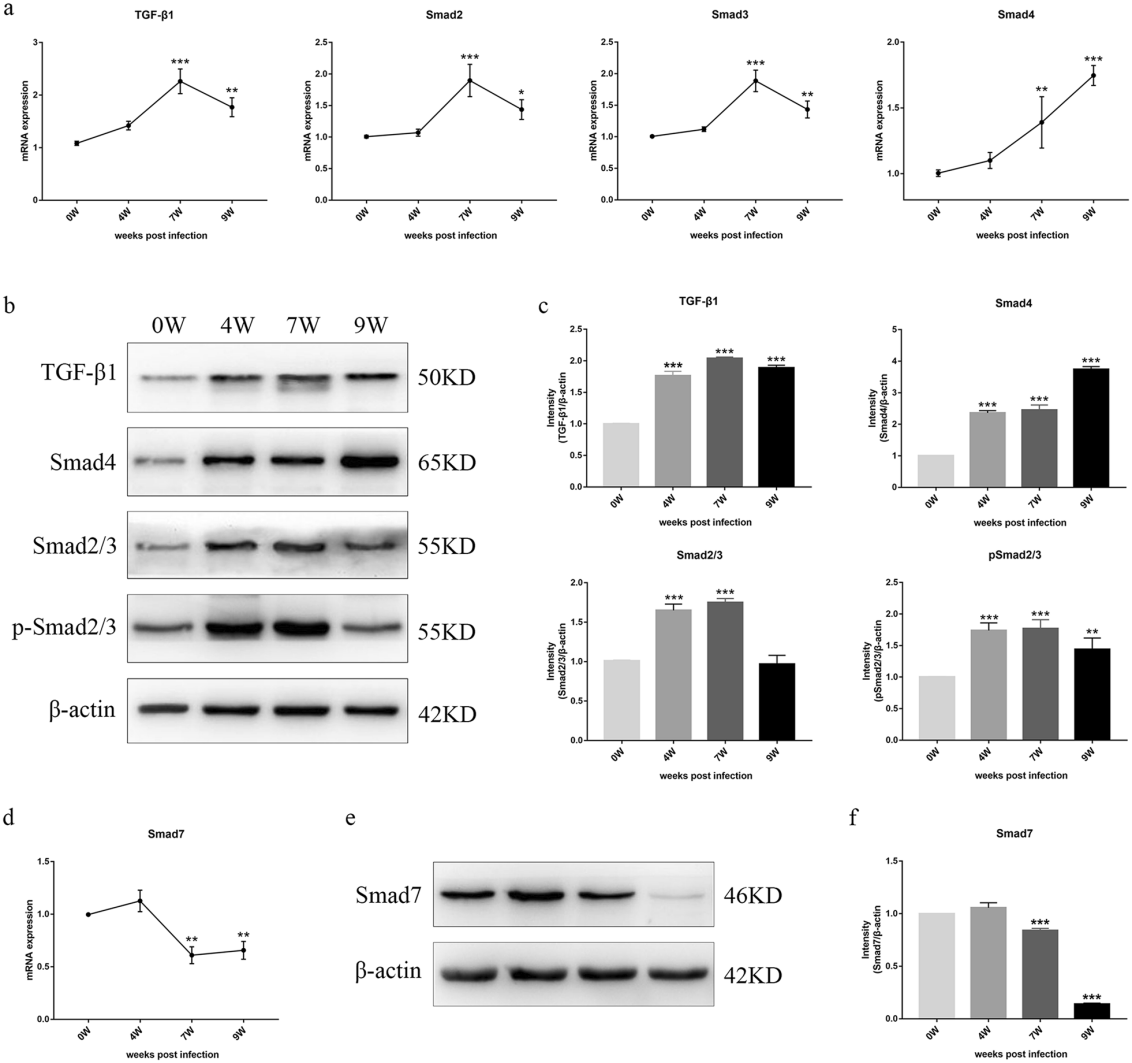
The effect of TGF-β1/Smad signaling in *S. japonicum*-infected mice. **a** The expression of the transcript of TGF-β1, Smad2/3, Smad4, and **d** Smad7 in primary HSCs at different time points was determined by RT-qPCR. **b** The expression of the protein of TGF-β1, Smad2/3, Smad4, and **e** Smad7 was detected by Western blot, **c**, **f**, and the densitometric analysis was normalized to the level of β-actin in primary HSCs. Data are presented as mean ± SD (*n* = 3). *P < 0.05, **P < 0.01, ***P < 0.001, compared with the 0 wpi group

To investigate the effect of TGF-β1/Smad signaling on HSC activation after *S. japonicum* infection, the mRNA and protein levels of important molecules, including TGF-β1, Smad2, Smad3, Smad4, and Smad7, in primary HSCs at different infection periods were measured. As shown in Fig. 3, the increased transcriptional levels of TGF-β1, Smad2, Smad3, and Smad4 after *S. japonicum* infection, and these levels peaked at 7 wpi (TGF-β1, *P* < 0.001; Smad2, *P* < 0.001; Smad3, *P* < 0.001) and 9 wpi (Smad4, *P* < 0.001), respectively. Otherwise, Smad7 mRNA expression showed no significant change at 4 wpi (*P* > 0.05) but strikingly decreased at 7 wpi (*P* < 0.001). These molecules were tested by Western blot to confirm this observation. The protein levels showed a correspondingly significant elevation of TGF-β1, Smad2/3, p-Smad2/3, and Smad4 increased as liver fibrosis progressed, and these mediators' levels peaked and had statistical significance at 7 wpi (TGF-β1: 2.04-fold, *P* < 0.001; Smad2/3: 1.75-fold, *P* < 0.001; p-Smad2/3: 1.77-fold, *P* < 0.001) and 9 wpi (Smad4: 3.74-fold, *P* < 0.001), respectively. Smad7, a negative regulator of TGF-β1/Smad signaling, was dramatically decreased at 9 wpi (*P* < 0.001). Thus, we inferred that primary HSCs might be regulated by TGF-β1/Smad signaling in schistosomiasis-infected mice and are involved in the process of liver fibrosis.

### Evaluation of miRNA expression in primary HSCs

**Fig. 4:**
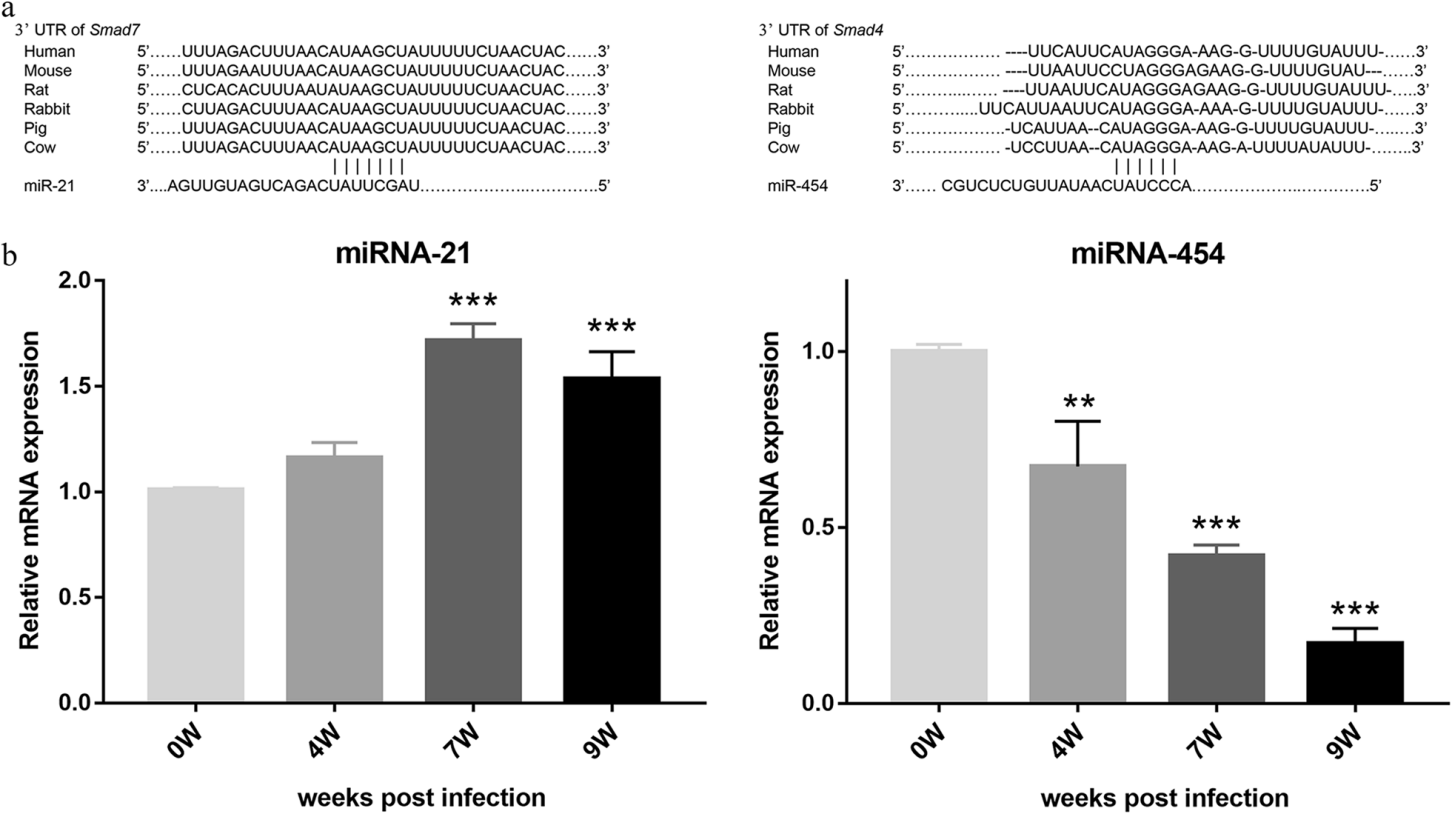
miRNA target prediction and the expression of miRNAs in primary HSCs of *S. japonicum*-infected mice at 0, 4, 7, and 9 weeks. **a** Bioinformatics analysis as predicted by TargetScan and miRanda showed that the 3′-UTR of Smad7 contained the binding site of miRNA-21, and the 3′-UTR of Smad4. **b** The relative expression levels of miRNA-21 and miRNA-454 were determined by RT-qPCR. Data are presented as mean ± SD (*n* = 3). *P < 0.01; ***P < 0.001, compared with the 0 wpi group

Potential targets of miRNAs were predicted by miRanda and TargetScan. The results demonstrated that the 3′-UTR of Smad7 contained the binding site of miRNA-21, and the 3'-UTR of Smad4 contained the binding site of miRNA-454, and both binding sites were highly conserved in many different species (Fig. 4a). We then examined the expression of miRNA-454 and miRNA-21 in primary HSCs of *S. japonicum*-infected mice by RT-qPCR. Unsurprisingly, the miRNA-21 expression showed no significant change at 4 wpi but was dramatically increased at 7 wpi (1.72-fold, *P* < 0.001) and decreased at 9 wpi, compared to the 0 wpi group. Meanwhile, the expression of miRNA-454 showed a decrease after parasite infection, reaching the lowest point at 9 wpi (*P* < 0.001). All data indicated that miRNAs also play a pivotal role in activating HSC and liver fibrosis after *S. japonicum* infection (Fig. 4b).

## Discussion

Liver fibrosis is a serious symptom of many chronic diseases associated with the morbidity and mortality of clinical patients. Many different cellular mechanisms are involved in the process of liver fibrosis, but HSCs seem to be the predominant cell type [23]. In an animal model of *S. japonicum*-infected mice, we found that the activation of HSCs contributed to liver fibrosis. Indeed, the activated HSCs facilitated the formation of inflammatory egg granulomas and liver fibrosis. Furthermore, we identified that the activation of HSCs was through TGF-β1/Smad signaling. Thereby, we revealed the role and regulatory mechanism of HSCs in schistosomiasis, which provided the scientific basis for finding targets for the prevention and treatment of liver fibrosis.

Many chronic inflammatory diseases, including hepatitis virus, helminth infections, NASH, and metabolic and autoimmune diseases, cause continuous hepatocellular damage leading to liver fibrosis [7–9]. Liver fibrosis can eventually lead to cirrhosis, which ultimately causes organ dysfunction and death. In developed countries, up to 45% of all deaths are attributable to pathological tissue remodeling [24]. Liver fibrosis is a complex process that requires cellular and extracellular signaling, including myofibroblasts, HSCs, hepatocytes, inflammatory cells, liver sinusoidal endothelial cells, portal fibroblasts, and fibrocytes [25]. Myofibroblasts are the main source of ECM in the fibrotic liver, which are activated in response to liver injury and are not present in the healthy liver [25, 26]. Previous research reported that liver-resident cells, HSCs, portal fibroblasts, fibrocytes, and mesenchymal stem cells could be conditionally transformed into liver myofibroblasts [25, 27–29]. In addition, activated HSCs and portal fibroblasts are the major sources of myofibroblasts, producing much of the collagen involved in the formation of liver fibrosis [28–30]. Due to the complex causes and pathogenesis of liver fibrosis, there is still no definitive treatment for clinical patients. Our study established a *S. japonicum*-infected mice model and detected the degree of liver fibrosis in mice at different time points. The results showed that egg granulomas and collagen fibers could be observed in the liver at 7 wpi, but there were no obvious pathological changes before 4 wpi. Notably, liver fibrosis became more severe in mice with the prolonged infection time.

Given the life history of *S. japonicum*, adult worms lay eggs in the portal system and mesenteric veins in the human body at 7 wpi, consistent with the point when we observed liver lesions in mice. It is indicated that the eggs were the main cause of pathological damage in the host after the *S. japonicum* infection. Although there have been many studies on the mechanism of HSC in schistosomiasis liver fibrosis in recent years [31–33], most of them were performed in vitro with the human HSC line (LX2) since primary HSCs were difficult to isolate. Therefore, our team used *S. japonicum-*infected FVB/NJ mice to isolate primary HSCs for in vitro culture to investigate the mechanism of liver fibrosis. Moreover, the purity of the isolated HSCs was over 90%, which was suitable for subsequent experiments. Emerging studies have demonstrated that high intracellular expression of α-SMA is a key marker of HSC activation, and collagen I and III are fibrotic markers [34, 35]. Our data displayed that the expression of α-SMA increased from the 4 weeks after *S. japonicum* infection and peaked at 9 wpi. Meanwhile, the expression of collagen I and collagen III was consistent with the trend of α-SMA, suggesting that the HSCs of *S. japonicum*-infected mice were significantly activated after 4 wpi, and the overexpression of collagen in the liver was the main factor causing liver fibrosis.

The classic mechanism of liver fibrosis is that liver injury triggers inflammatory responses, causing the activation of macrophages to release reactive oxygen species and TGF-β1. Then, quiescent HSCs activated and transformed into myofibroblasts in response to TGF-β1 in Smads-dependent and/or Smads-independent manner. Finally, the activated HSC produces a large amount of collagen, which leads to excessive ECM deposition and liver fibrosis [14, 25]. Once the cause of liver injury is removed, myofibroblasts undergo apoptosis and ameliorate the progression of liver fibrosis. Thus, therapeutic agents designed to block and reverse lesion changes could effectively accelerate the regression of liver fibrosis. Recent studies demonstrated that in a rat model of carbon tetrachloride-induced liver fibrosis, the expression of p-Smad2 and p-Smad3 in the liver was significantly increased [36]. In addition, our team has previously clarified that TGF-β1, p-Smad2, and p-Smad3 were enhanced in the liver of *S. japonicum*-infected mice at 7 wpi [6]. Consistent with the previous results, this paper revealed that the TGF-β1/Smad signaling was activated in HSCs from 4 wpi. The expression of TGF-β1, Smad2/3, p-Smad2/3, and Smad4 sharply increased as liver fibrosis progressed and peaked at 7 or 9 wpi, while the negative regulator Smad7 dramatically decreased at 9 wpi. With the development of molecular biology, growing evidence has indicated that miRNAs are involved in various signal transduction processes, including inflammation, cell proliferation, apoptosis, and fibrosis, and mediate the occurrence and development of various diseases. Some miRNAs are associated with liver fibrosis, such as miR-21, miR-221/222, and miR-181b, which promote liver fibrosis through TGF-β and NF-κB signaling. However, miR-29b, miR-101, miR-122, and miR-214–3 prevent fibrosis by inhibiting the TGF-β pathway and collagenase synthesis [37].

Moreover, previous reports have suggested that downregulation of miRNA-21 in HSCs alleviates liver fibrosis through overexpression of Smad7, and miRNA-454 directly targets Smad4 to inhibit the activation of HSCs [18, 19]. Bioinformatics results revealed that the 3′UTR of Smad4 and smad7 contained binding sites for miR-21 and -454, respectively, and that this region showed highly conserved sequences in many different species, including humans and mice, suggesting that the mechanism of miRNAs regulating Smads might be species-independent. Moreover, we found that miRNA-21 and miRNA-454 participated in the activation of HSCs during *S. japonicum* infection, in which the expression of miRNA-21 increased after 7 wpi and miRNA-454 decreased after parasite infection. Interestingly, the expression of Smad7 dramatically decreased after 7 wpi, whereas Smad4 continued to increase after infection. These data imply that miRNAs can regulate schistosomiasis liver fibrosis through Smad proteins, although the specific regulatory mechanism needs further study. Hence, our study revealed that TGF-β1/Smad signaling plays a critical role in the activation of HSCs and liver fibrosis after *S. japonicum* infection.

## Conclusions

Altogether, our study indicated that HSCs mediate the occurrence and development of liver fibrosis in schistosomiasis and are regulated by the TGF-β1/Smad signaling pathway. These functions of HSCs played a vital role in liver fibrosis induced by *S. japonicum* infection. Our study provides a theoretical basis for the prevention and control of schistosomiasis through a deeper understanding of the mechanism of liver fibrosis in schistosomiasis.

## Supplementary Information

**Additional file 1: Figure S1.** The isolation, identification, and culture of primary HSCs in *S. japonicum*-infected mice. (a–c) Cell autofluorescence was observed under an inverted fluorescence microscope at a light wavelength of 328 nm. (d–f) The expression of GFAP in primary HSCs in *S. japonicum*-infected mice was examined by immunocytochemical staining. (g–i) The growth status of primary HSCs in culture for 0, 5, and 7 days.

## Data Availability

All data supporting the conclusions of this study are included in the article.

## Abbreviations

*S. japonicum*: *Schistosoma japonicum*
HSCs: Hepatic stellate cells
WHO: World Health Organization
TGF-β1: Transforming growth factor-β1
α-SMA: Alpha-smooth muscle actin
ECM: Extracellular matrix
miRNAs: MicroRNAs
3′ UTR: 3′ Untranslated region
*O. hupensis*: *Oncomelania hupensis*
H&E: Hematoxylin–eosin
RT-qPCR: Quantitative real-time polymerase chain reaction
Mean ± SD: Mean ± standard deviation
wpi: Weeks post-infection
