# Supplementary figures and images for: Activation of primary hepatic stellate cells and liver fibrosis induced by targeting TGF-β1/Smad signaling in schistosomiasis in mice
Source: Parasit Vectors. 2022 Dec 6;15:456. doi: 10.1186/s13071-022-05584-1 (PMC9727849; doi:10.1186/s13071-022-05584-1)

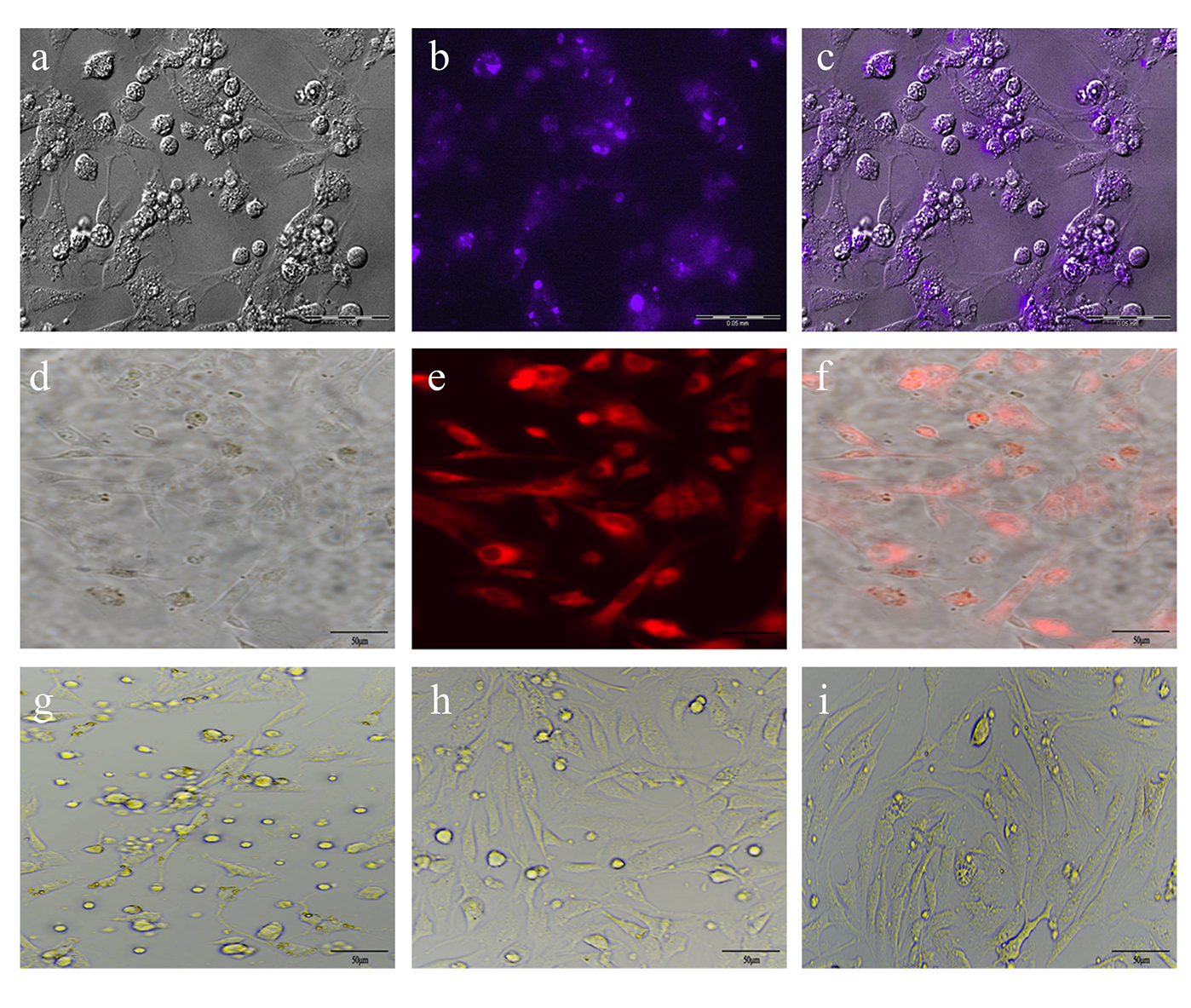

Supplement: Supplementary file 1 — Additional file 1: Figure S1. The isolation, identification, and culture of primary HSCs in S. japonicum-infected mice. (a–c) Cell autofluorescence was observed under an inverted fluorescence microscope at a light wavelength of 328 nm. (d–f) The expression of GFAP in primary HSCs in S. japonicum-infected mice was examined by immunocytochemical staining. (g–i) The growth status of primary HSCs in culture for 0, 5, and 7 days. [file 13071_2022_5584_MOESM1_ESM.tif]
